# Probiotic *Lactobacillus rhamnosus GR-1* is a unique prophylactic agent that suppresses infection-induced myometrial cell responses

**DOI:** 10.1038/s41598-019-41133-0

**Published:** 2019-03-18

**Authors:** Bona Kim, Oksana Shynlova, Stephen Lye

**Affiliations:** 1 Department of Physiology, University of Toronto, Toronto, Canada; 2 Department of Obstetrics and Gynaecology, University of Toronto, Toronto, Canada; 3 Lunenfeld-Tanenbaum Research Institute, Sinai Health System, Toronto, Canada

## Abstract

Preterm birth (PTB) is a multifactorial syndrome affecting millions of neonates worldwide. Intrauterine infection can induce PTB through the secretion of pro-inflammatory cytokines and untimely activation of uterine contractions. In pregnant mice, prophylactic administration of probiotic *Lactobacillus rhamnosus GR-1* supernatant (GR1SN) prevented lipopolysaccharide (LPS)-induced PTB and reduced cytokine expression in the uterine muscle (myometrium). In this study we sought to delineate the mechanisms by which GR1SN suppressed cytokine secretion in the myometrium. We observed that *L*. *rhamnosus GR-1* uniquely secretes heat-resistant but trypsin-sensitive factors, which significantly suppressed LPS-induced secretion of pro-inflammatory cytokines IL-6, IL-8, and MCP-1 in the human myometrial cell line, hTERT-HM. This effect was unique to GR1SN and could not be replicated using supernatant derived from non-*GR-1* commensal lactobacilli species: *L*. *rhamnosus GG*, *L*. *lactis*, *L*. *casei*, or *L*. *reuteri RC-14*. Furthermore, pre-incubation of hTERT-HM cells with low-dose Pam3CSK (a TLR1/2 synthetic agonist which mimics LPS action) prior to LPS administration also significantly decreased LPS-induced cytokine secretion. This study highlights the distinct capacity of protein-like moieties secreted by *L*. *rhamnosus GR-1* to inhibit pro-inflammatory cytokine production by human myometrial cells, potentially through a TLR1/2-mediated mechanism.

## Introduction

Preterm birth (PTB; birth of an infant prior to 37 weeks of gestation) is a global medical concern that affects 13 million mothers and infants, annually. PTB is a syndrome with multiple etiologies. Approximately, one third of PTBs results from medically induced preterm labor (PTL), associated with gestational complications such as preeclampsia or intrauterine growth restriction^1^, while another third results from idiopathic spontaneous PTL with or without rupture of the membranes^2^. The remaining 40% of PTBs are associated with intrauterine infection (IUI)^1^, commonly caused by ascending urogenital tract infections, or systemic maternal infections. Despite clinical intervention to treat IUI in pregnant women with antibiotics, a recent meta-analysis reported limited effectiveness of such therapy in preventing or delaying PTL^3^.

Interestingly, clinical studies have reported an association between vaginal dysbiosis and increased risk of acquiring IUI. A population study found that in healthy, non-pregnant women, the lack of a dominant *lactobacilli* species in the vaginal microbiome was associated with increased abundance of pathogenic bacteria, higher vaginal pH, and higher Nugent scores (a classical diagnostic scoring system for rod-shaped bacteria)^4^, predisposing women to an increased risk of acquiring sexually transmitted diseases and common gynecological complications, such as bacterial vaginosis or vaginal candidiasis^5^. The vaginal microbiome during pregnancy is dominated by *lactobacilli* species due to increased estrogen metabolites which serve as nutrients for *lactobacilli*, and consequentially heightens protection from pathogens. Although some studies have reported no direct link between vaginal bacterial composition during pregnancy and rates of PTB^6^, others have observed the presence of certain *lactobacilli* species in a low-risk of PTB cohort, while a higher presence of pathogenic bacteria subspecies in the vaginal microbiome was associated with PTB^7^.

*L*. *rhamnosus GR-1*, a strain of commensal vaginal microbiota has been investigated as a potential probiotic species. Prophylactic administration of *GR-1* supernatant (GR1SN) to pregnant mice resulted in significant delay in lipopolysaccharide (LPS)-induced PTB which was associated with reduced expression of multiple pro-inflammatory cytokines IL-6, TNF-α, CSF-2, IL-3, IL-9, IL-12, IL-13, and IL-17 by pregnant uterine tissues, including the myometrial smooth muscle layer (Yang *et al*., 2014). *In vitro*, GR1SN was able to inhibit cytokine secretion following LPS exposure in primary human trophoblasts^8,9^, decidua^10^, and amniotic cells^11^. Similarly, *in vitro* GR1SN exposure to human macrophages resulted in increased expression of granulocyte-colony stimulating factor (G-CSF/CSF3) and reduced secretion of the pro-inflammatory cytokine, tumor-necrosis factor (TNF-α)^12^. Further investigation indicated that G-CSF induction by GR1SN in the absence of TNF-α is due to activation of a toll-like receptor (TLR)2-dependent pathway^13^. TLR2, a membrane-bound receptor for bacterial lipoproteins, signals activation of inflammatory pathways, much like TLR4, the receptor for endotoxin (LPS). The two pathways share numerous downstream signalling molecules and have been implicated in regulating endotoxin tolerance^14^, a mechanism by which cells become less responsive to a secondary endotoxin stimulus (i.e. LPS) following pre-treatment with low-doses of identical or similar agonists. Increased expression of TLR2 and TLR4 has been observed in the cervix^15^ and the decidua in cases of infectious PTB^16^, and spontaneous PTL^17^. In the human myometrium, TLR2 and TLR4 mRNA and protein are increased during term compared to preterm labor, and TLR2 is highly expressed during labour compared to term non-labour^18^. TLR4 signalling has been implicated in the initiation and regulation of parturition. For example, in TLR4-mutant mice LPS failed to induce PTB^19^.

The myometrium is an immune-modulatory tissue, capable of producing cytokines^20^. Premature induction of pro-inflammatory cytokines, including TNF-α and IL-6, and chemokines such as IL-8 and MCP-1 by local or systemic infection can initiate pro-labour pathways, through increased expression of uterotonins (eg. prostaglandins), and contraction-associated proteins^21–23^. In addition, chemokines are shown to stimulate recruitment of maternal peripheral leukocytes into uterine tissues, simultaneously amplifying the inflammatory signals. Conversely, anti-inflammatory cytokines restrict pro-inflammatory pathways, limiting the adverse effects of a chronic or acute uterine inflammation. Thus, TLR pathways, which regulate cytokine and chemokine activation, offer promising targets for the prevention of infection-mediated PTB^24^. We hypothesized that GR1SN contains factors secreted by probiotic *Lactobacillus rhamnosus GR-1* which inhibit pro-inflammatory cytokine expression in the myometrium. Here, using a human myometrial cell line (hTERT-HM), we examined *in vitro* whether GR1SN can abrogate the LPS-induced inflammatory response mediated through TLR signalling.

## Results

### Effect of LPS on cytokine production by human myometrial cells

**Figure 1:**
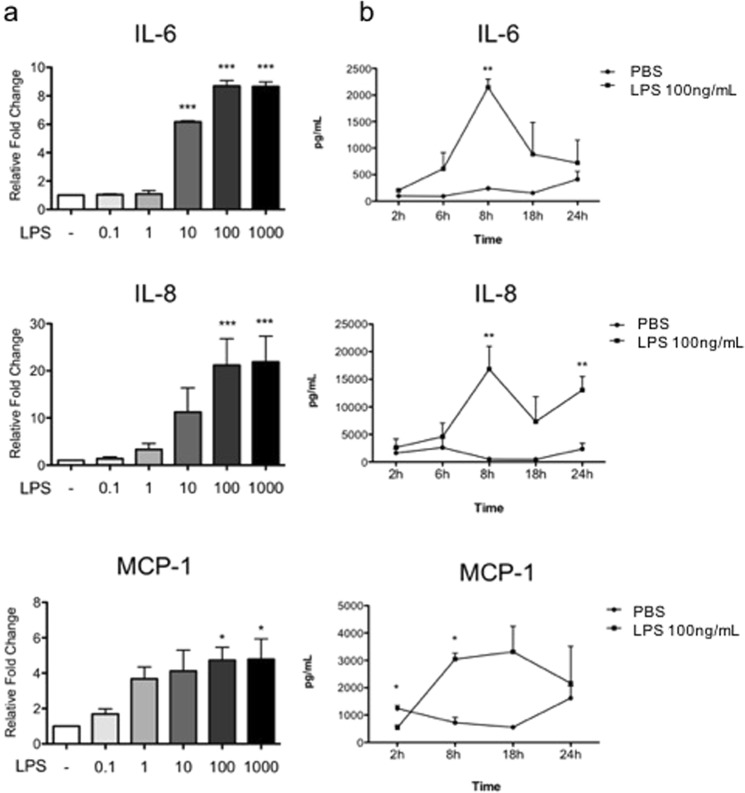
Comparative dose and time response following LPS administration in hTERT-HM cells. (**a**) Cells were treated for 8 hours with various doses of LPS (0.1–1000 ng/mL, grey to black bars) or vehicle (PBS, white bars). Statistical analysis was performed using One-way ANOVA followed by Dunnett post-test and presented as fold change (mean ± SEM) relative to untreated control. Values were considered statistically significant when *P* < 0.05. (**b**) Cells were treated with 100 ng/mL of LPS for various time points. Data are shown as absolute concentrations [pg/mL]. Significance was determined by t-tests between LPS and PBS (vehicle control). A significant difference between LPS and vehicle is indicated by *. *P < 0.05, **P < 0.01, ***P < 0.001 (n = 7).

Primary human myometrial cells were exposed to LPS (100 ng/mL) for 8 hours to induce an immune response (n = 5). Media conditioned by primary myometrial cells was found to contain elevated levels of three cytokines (IL-6, IL-8, and MCP-1) (Supplemental Fig. 1) similar to previously published reports in human trophoblasts^8^ and decidual cells^10^. However, because of high patient-to-patient sample variation in cytokine expression, we chose to use a human myometrial cell line (hTERT-HM) for the current study. hTERT-HM cells stimulated with LPS (100 ng/mL) secreted levels of IL-6, IL-8, and MCP-1 proteins (Fig. 1, n = 7) that were comparable to levels secreted by primary myometrial cells.

### GR1SN-MRS pre-treatment attenuates LPS-induced cytokine and chemokine secretion by hTERT-HM cells

**Figure 2:**
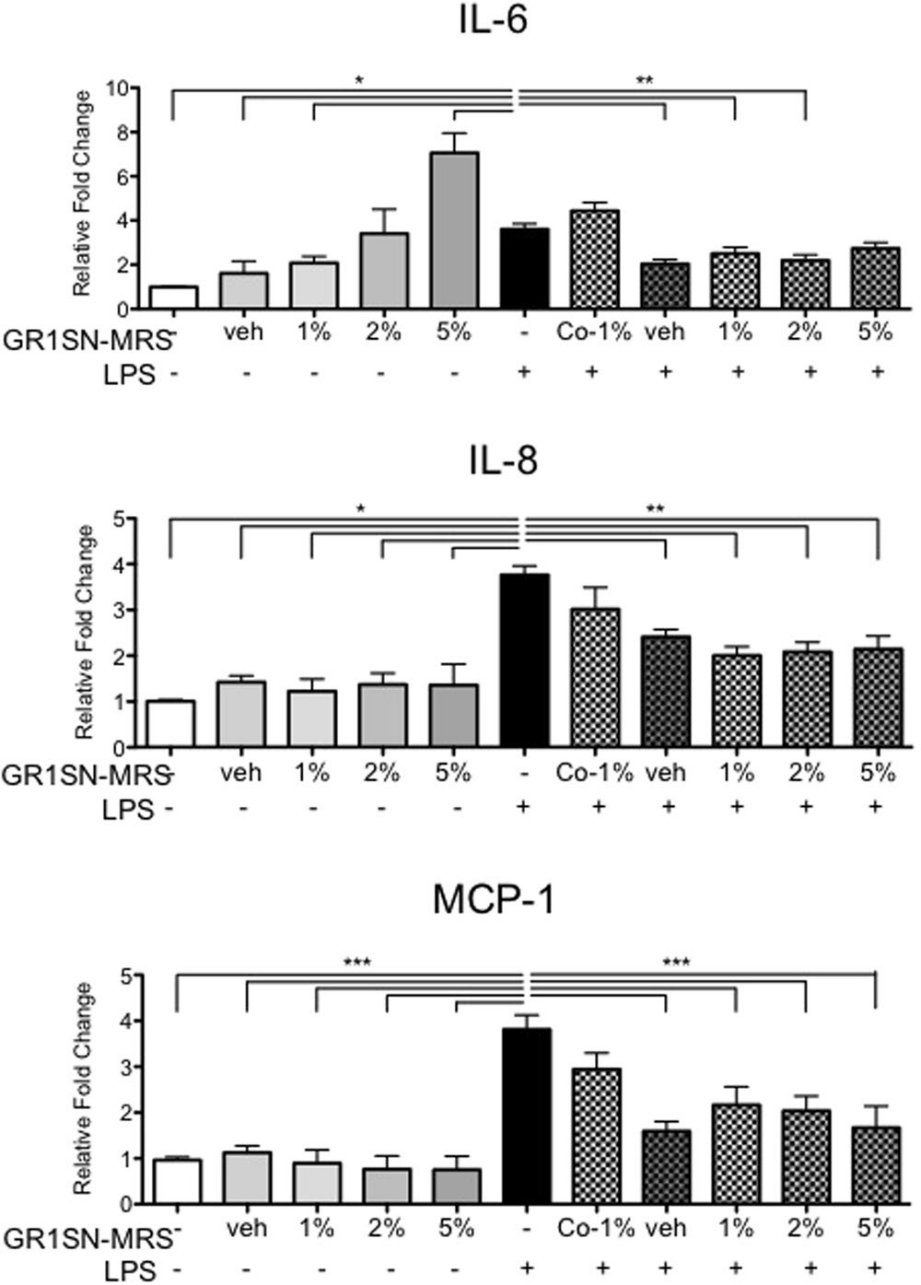
Effect of GR1SN-MRS treatment on hTERT-HM cell cytokine secretion following LPS stimulus. hTERT-HM cells were treated with MRS broth (1% v/v) or various doses of GR1SN-MRS (1, 2, 5% v/v) without LPS stimulus (solid grey bars), for 16 hours prior to (checkered grey bars), or concurrently with (GR1SN-MRS 1%, v/v, “Co-1%”) LPS (100 ng/mL). Values are presented as fold change (mean ± SEM) relative to untreated control (solid white bars). Statistical significance was determined by One-way ANOVA followed by Dunnett’s post-test relative to LPS stimulus (solid black bar). *P < 0.05; **P < 0.01; ***P < 0.001 (n = 9).

hTERT-HM cells were pre-treated with 1, 2, or 5% v/v GR1SN-MRS, with or without LPS (100 ng/mL). Pre-treatment of hTERT-HM cells with low dose GR1SN-MRS (1% v/v) for 16 hours prior to LPS significantly suppressed secretions of IL-6 (31%, P < 0.01), IL-8 (54%, P < 0.001), and MCP-1 (65.5%, P < 0.001) compared to cells exposed to LPS only (Fig. 2, n = 9). Conversely, concurrent treatment of cells with GR1SN-MRS and LPS did not result in suppression of IL-6, IL-8, or MCP-1 secretion. Interestingly, exposure of hTERT-HM cells to GR1SN-MRS alone for 24 hours significantly induced IL-6 secretion in a dose-dependent manner compared to untreated cells (5% v/v, 7-fold increase). IL-8 and MCP-1 secretions were not affected by exposure to GR1SN-MRS.

### MRS broth alone impairs LPS-induced myometrial inflammation

**Figure 3:**
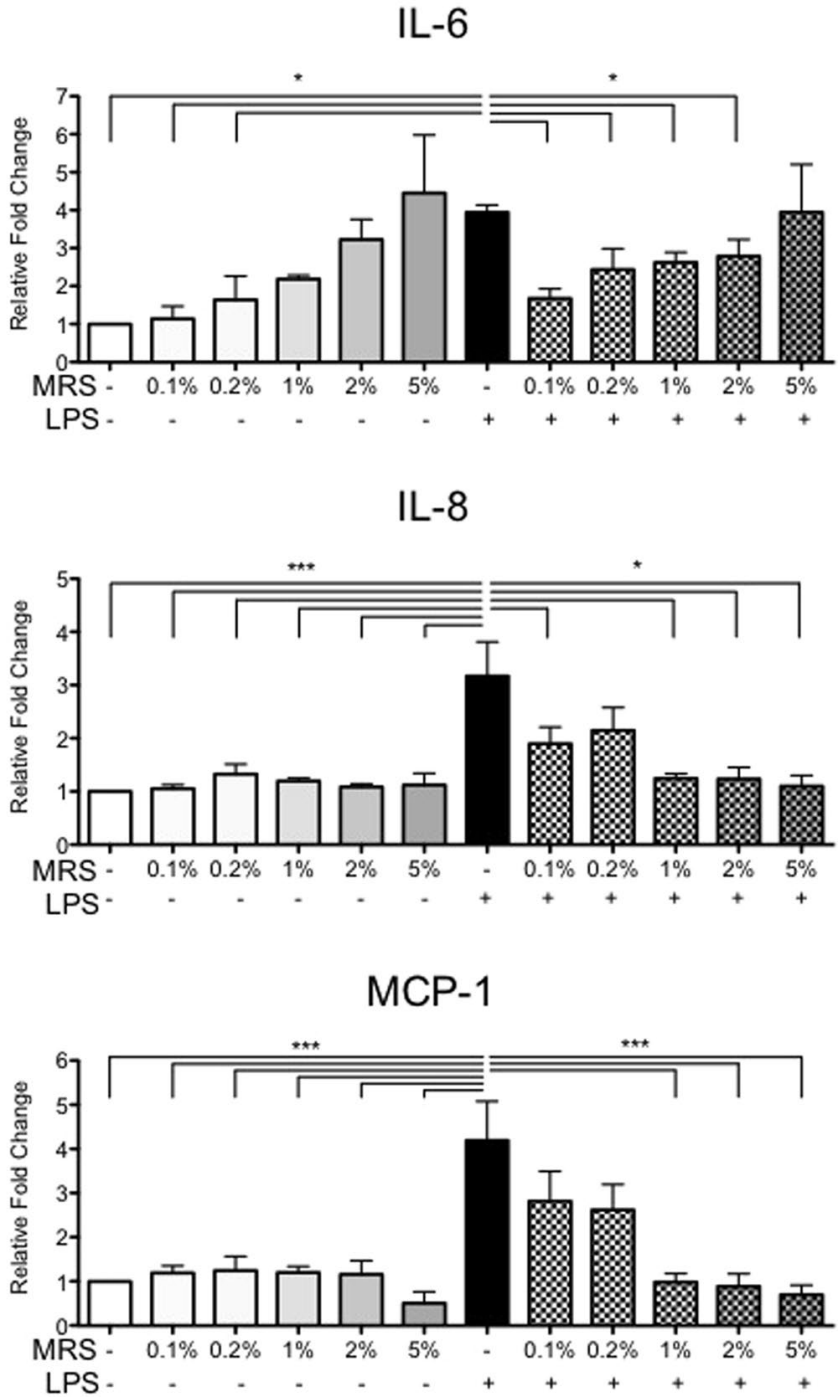
Effect of MRS broth on cytokine secretion in hTERT-HM. hTERT-HM cells were treated with various doses of MRS broth (0.1–5% v/v) without LPS stimulus (solid grey bars) or for 16 hours prior to (checkered grey bars). Values are presented as fold change (mean ± SEM) relative to untreated control (solid white bars). Statistical significance was determined by One-way ANOVA followed by Dunnett’s post-test relative to LPS stimulus (100 ng/mL, solid black bar). *P < 0.05; **P < 0.01; ***P < 0.001 (n = 4).

We sought to distinguish the effect of the GR1SN-MRS from MRS alone on LPS-induced inflammatory cytokines. Cells were cultured with increasing doses of MRS broth in culture media (0.1, 0.5, 1, 5% v/v). ELISA demonstrated that, similar to the effects of GR1SN-MRS, MRS treatment alone (5% v/v) significantly induced IL-6 concentrations in conditioned media (CM) relative to untreated cells (4.5-fold increase, p < 0.05) (Fig. 3, n = 4). Secretion of IL-8 and MCP-1 were not affected by MRS broth. Surprisingly, when 0.1% v/v MRS broth was administered prior to LPS stimulus (100 ng/mL), a significant reduction of IL-6 secretion (57.6%, P < 0.05) was observed, while pre-treatment at 1%, 2%, 5% v/v MRS resulted in significant (p < 0.001) suppression of LPS-induced MCP-1 and IL-8 (Fig. 3).

### GR1SN-PBS pre-treatment suppresses LPS-induced cytokine secretions by hTERT-HM cells

**Figure 4:**
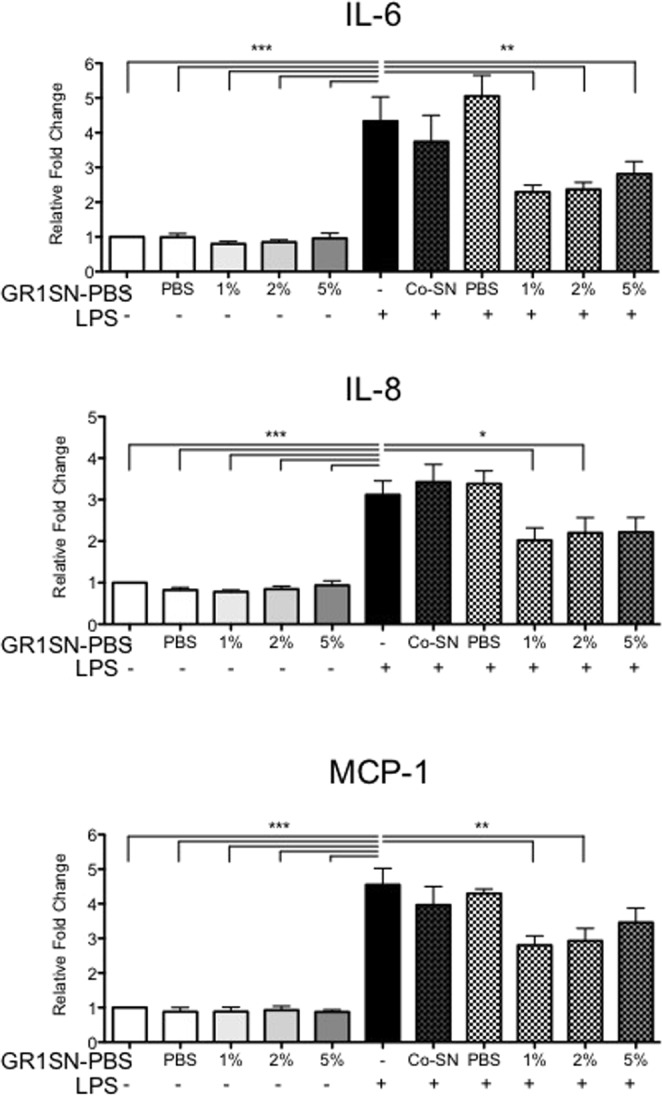
Effect of GR1SN-PBS treatment on hTERT-HM cell cytokine secretion following LPS stimulus. hTERT-HM cells were treated with PBS (1% v/v, “PBS”) or various doses of GR1SN-PBS (1, 2, 5% v/v) without LPS stimulus (solid grey bars), for 16 hours prior to (checkered grey bars), or concurrently with (GR1SN-PBS 1%, v/v, “Co-1%”) LPS (100 ng/mL). Values are presented as fold change (mean ± SEM) relative to untreated control (solid white bars). Statistical significance was determined by One-way ANOVA followed by Dunnett’s post-test relative to LPS stimulus (solid black bar). *P < 0.05; **P < 0.01; ***P < 0.001 (n = 9).

Following observations that MRS broth alone can affect cytokine secretion by hTERT-HM cells, PBS-based GR1SN-PBS was developed to remove MRS broth components and examine the action of *L*. *rhamnosus GR-1* secreted molecules, alone. Myometrial cells were treated with GR1SN-PBS, CM was collected and analyzed by ELISA. GR1SN-PBS alone did not induce secretion of IL-6, IL-8, or MCP-1 by myometrial cells, however pre-treatment with GR1SN-PBS for 16 hours prior to LPS (100 ng/mL) resulted in significant (p < 0.05) suppression of IL-6 by 47.11%, IL-8 by 34.8% and of MCP-1 by 38.4% as compared to cells stimulated with LPS alone (Fig. 4, n = 9). Concurrent treatment of cells with GR1SN-PBS and LPS (100 ng/ml) did not inhibit cytokine secretions by myometrial cells in response to LPS stimulus.

### Potential mechanism of GR1SN action on hTERT-HM cells

**Figure 5:**
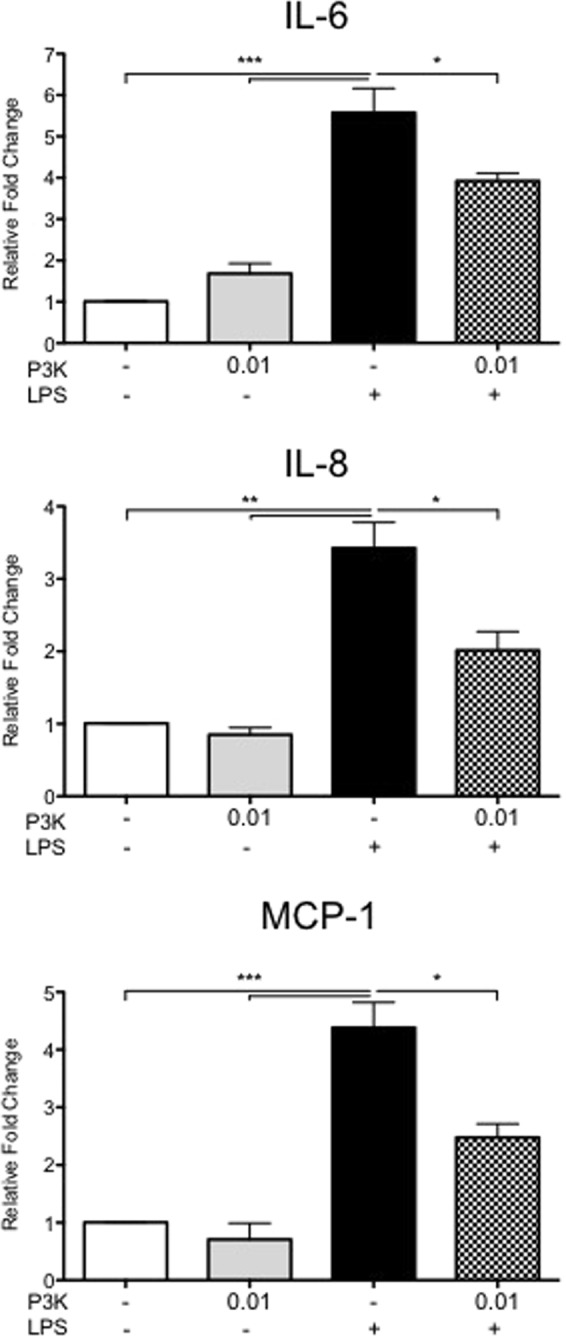
Effect of low-dose Pam3CSK pre-treatment prior to high-dose LPS stimulus on cytokine secretions by hTERT-HM cells. Cells were pre-treated with 0.01 μg/mL of Pam3CSK (P3K) with (checkered bars) or without (solid grey bars) LPS stimulus (100 ng/mL). Values are presented as fold change (mean ± SEM) relative to untreated control (white bars). Statistical significance was determined by One-way ANOVA followed by Dunnett’s post-test relative to LPS stimulus (solid black bars). *P < 0.05; **P < 0.01; ***P < 0.001 (n = 5).

**Figure 6:**
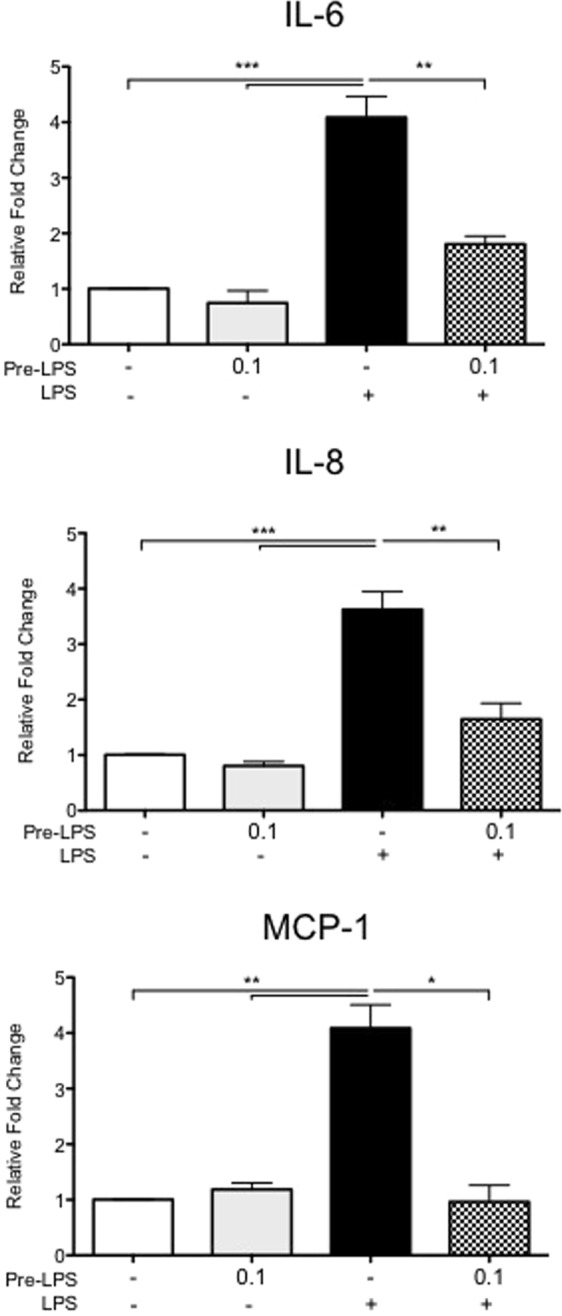
Effect of low-dose LPS pre-treatment prior to high-dose LPS stimulus on cytokine secretions by hTERT-HM cells. Cells were pre-treated with 0.1 μg/mL of LPS prior to a subsequent treated with (checkered grey bars) or without (solid grey bars) LPS (100 ng/mL). Values are presented as fold change (mean ± SEM) relative to untreated control (white bars). Statistical significance was determined by One-way ANOVA followed by Dunnett’s post-test relative to LPS stimulus (solid black bars). *P < 0.05; **P < 0.01; ***P < 0.001 (n = 5).

As TLR pathways regulate cytokine activation, and pre-incubation of cells with GR1SN-PBS influences LPS-induced cytokine secretions, we hypothesized that active components within GR1SN might be TLR ligands. To confirm if cytokine secretion can be modulated by altering TLR pathways, we first treated hTERT-HM cells with Pam3CSK4 (a synthetic agonist for TLR1/2 that mimics bacterial lipoprotein expressed by *Lactobacilli* species), prior to LPS stimulus. Pam3CSK4 pre-treatment suppressed LPS-induced secretions of IL-6 by 38% (P < 0.01), IL-8 by 41% (P < 0.05), and MCP-1 by 43% (P < 0.05) as compared to LPS stimulus alone (Fig. 5, n = 5). To determine if “endotoxin tolerance”, might explain reduced cellular response to repeated stimuli, hTERT-HM cells were pre-treated with non-stimulatory doses (0.1 ng/mL) of LPS (TLR4 agonist) followed by a stimulatory dose of LPS (100 ng/mL, Fig. 1). As shown in Fig. 6 (n = 5) prior exposure to the TLR4 agonist significantly reduced LPS induced expression of IL-6 by 43.5% (P < 0.05), IL-8 by 38.9% (P < 0.05), and MCP-1 by 31% (P < 0.01).

### Identifying an active component of *GR-1* bacteria supernatant

**Figure 7:**
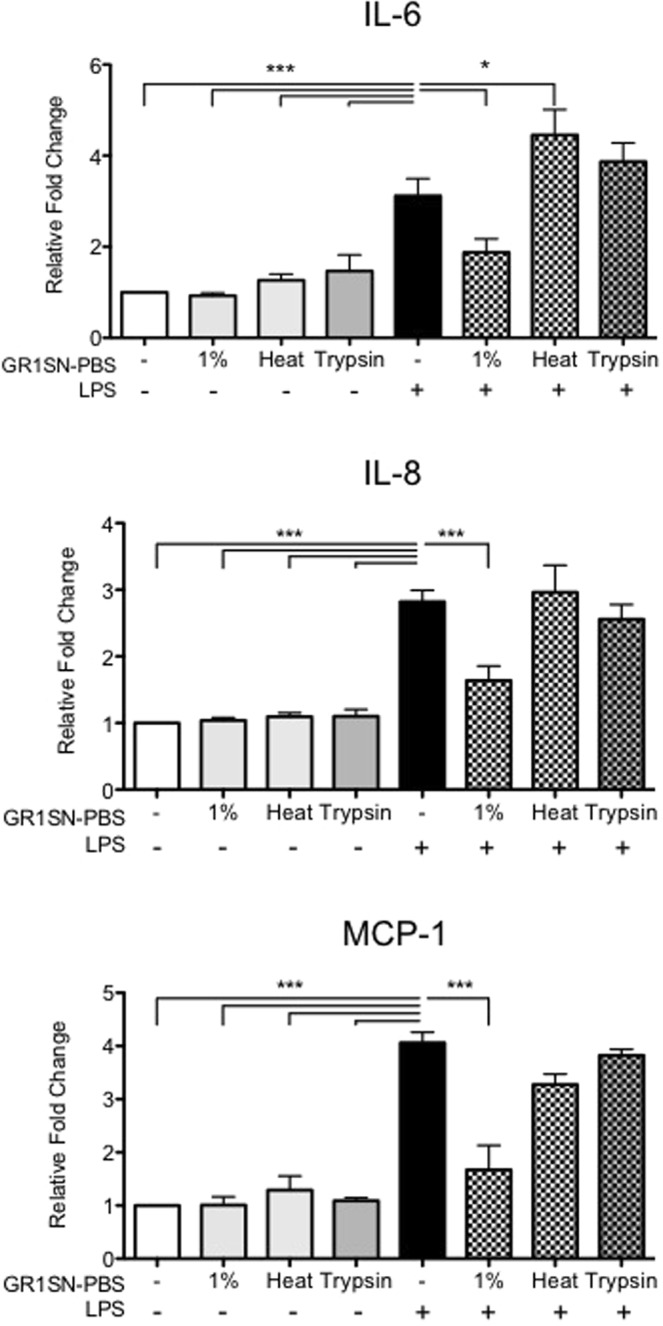
Effect of modified GR1SN-PBS on LPS-induced cytokine secretions. hTERT-HM cells were treated with non-modified GR1SN-PBS (“1%”), or with GR1SN-PBS modified to denature protein structures (“Heat”) or to degrade proteins (“Trypsin”), with (checkered grey bars) or without (solid grey bars) LPS stimulus (100 ng/mL). Values are presented as fold change (mean ± SEM) relative to untreated cells (white bars). Statistical significance was determined by One-way ANOVA followed by Dunnett’s post-test relative to LPS stimulus (solid black bars). *P < 0.05; **P < 0.01; ***P < 0.001 (n = 4).

**Figure 8:**
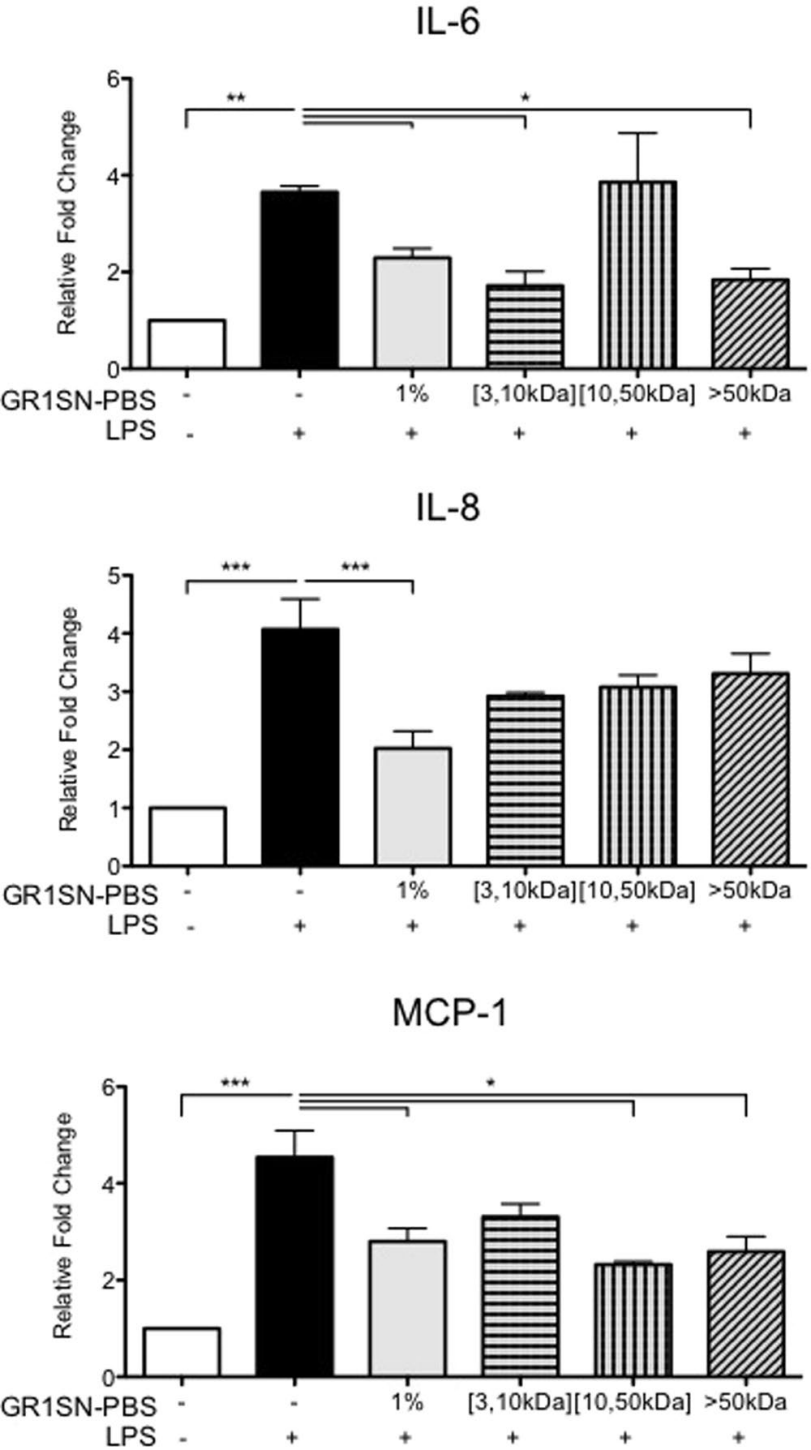
Effect of hTERT-HM pre-treatment with various GR1SN-PBS size fractions on LPS-induced cytokine secretions. hTERT-HM cells were pre-treated with non-modified GR1SN-PBS (solid grey bar, “1%”), or with size-fractioned GR1SN-PBS (striped bars) 16 hours prior to LPS stimulus (100 ng/mL). GR1SN-PBS was separated into fractions between 3 and 10 kDa ([3,10 kDa]), between 10 and 50 kDa ([10,50 kDa]), or greater than 50 kDa (>50 kDa). Values are presented as fold change (mean ± SEM) relative to untreated control (white bars). Statistical significance was determined by One-way ANOVA followed by Dunnett’s post-test relative to LPS stimulus (solid black bars). *P < 0.05; **P < 0.01; ***P < 0.001 (n = 3).

Next, we sought to identify the active components within GR1SN. As it was recently published that *GR-1* bacteria can secrete protein-like factors capable of regulating immune signalling pathways in human monocytes^13^, we analyzed protein content of GR1SN-PBS and found a total protein concentration of 22 µg/mL. To verify that the immunomodulatory activity of GR1SN-PBS was provided by protein, we modified the SN by either heat treatment (30 min at 95 °C) or trypsin incubation (1:1 v/v for 20 min at 37 °C) to remove functional protein activity. Both modifications of GR1SN-PBS fully ablated the suppressive effect of LPS on hTERT-HM cytokine expression, suggesting involvement protein/lipoprotein factors produced by *GR-1* bacteria (Fig. 7, n = 4). In an effort to isolate the protein moieties of GR1SN-PBS by size, the supernatant was centrifuged using filtration tubes at three different membrane sizes: 3 kDa, 10 kDa, 50 kDa. The amount of protein within each fraction was quantified by BCA analysis. From the initial 22 µg/mL of total protein, 4.24 µg/mL was found in the >50 kDa fraction, and 13 µg/mL was found in the <50 kDa fraction. All the lower molecular weight proteins (≈13 µg/mL) were found in the filtrate of 10 kDa and 3 kDa. Pre-treatment of hTERT-HM cells with the >50 kDa fraction resulted in significant suppression by 50.4% (P < 0.001) of MCP-1 only; while pre-treatment with all three low molecular weight fractions (<50 kDa, <10 kDa and <3 kDa fraction), were able to significantly (P < 0.01) suppress secretion of IL-6 by 32.3%, IL-8 by 53.5%, and MCP-1 by 50%, compared to cells exposed to LPS only (Fig. 8, n = 3).

### *L*. *rhamnosus GR-1* SN is a unique suppressor of LPS-induced cytokine secretion by hTERT-HM cells

**Figure 9:**
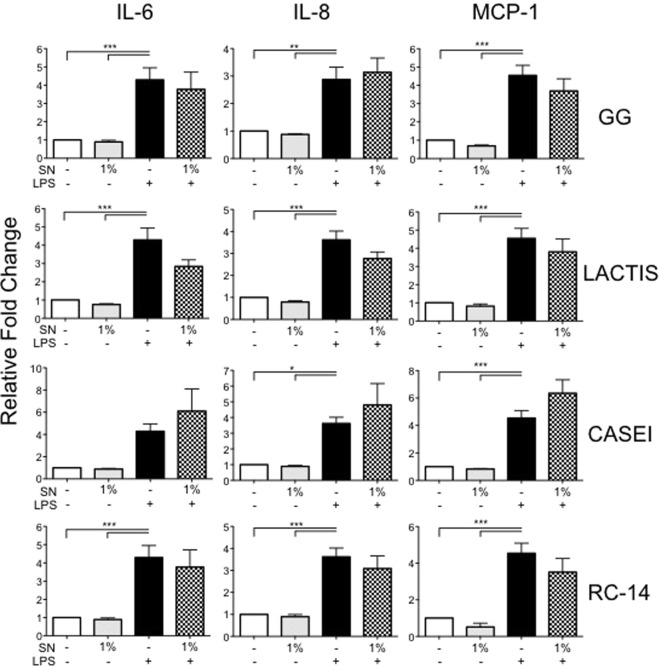
Effect of four different lactobacilli strains on cytokine suppression in hTERT-HM cells stimulated with LPS. Cells were pre-treated with 1% v/v of distinct Lactobacilli species with (checkered) or without (solid) LPS stimulus (100 ng/mL). Results are demonstrated as fold change (mean ± SEM) compared to untreated control (white bars). Statistical significance was determined by One-way ANOVA followed by Dunnett’s post-test compared to LPS stimulus (solid black bars) performed with all doses (1%, 2%, 5%). GG, L *rhamnosus GG*; Lactis, L. *lactis*; Casei, L. *casei*; RC-14, L. *reuteri RC-14*. *P < 0.05; **P < 0.01; ***P < 0.001 (n = 9).

To determine if immunomodulatory effect of *L*. *rhamnosus* was ubiquitous across other *lactobacilli* species, hTERT-HM cells were pre-treated with PBS-based SN collected from four other *lactobacilli* strains, commensal to diverse tissue types (L. *rhamnosus GG*, L. *lactis*, L. *casei*, and L. *reuteri RC-14)* following a protocol identical to that described for GR1SN. ELISA of CM from differentially treated hTERT-HM cells demonstrated that SN from *L*. *rhamnosus GG*, *L*. *lactis*, *L*. *casei*, and *L*. *reuteri RC-14* did not impact LPS-induced cytokine secretion by hTERT-HM cells (Fig. 9, n = 9) indicating the specificity of these effects to L. *rhamnosus GR-1*.

## Discussion

In this study we demonstrated the presence of low molecular weight, protein-like components in secretions of L. rhamnosus GR-1 that are capable of suppressing LPS-induced pro-inflammatory cytokine secretion by human myometrial cells. To date, Lactobacilli-secreted factors have been studied using De Man, Rogosa, and Sharpe (MRS) broth, an optimized growth media for Lactobacilli species, which contains a variety of growth factors (including approximately 2000 μg/mL of proteins detected by a BCA assay in this study - data not shown). Such factors include beef and yeast extracts, the precise composition of which is not disclosed by the manufacturer (BD Bioscience Inc), but could interact with *in vivo* and *in vitro* environments to influence intracellular signals. Other components include factors that help selective proliferation of lactobacilli strains such as polysorbate 80, sodium acetate, and magnesium sulphate, which are ubiquitously used in food preparation, medical, and laboratory settings. These factors have been shown in various *in vitro* and *in vivo* systems to alter expression of cytokines including IL-6, IL-1β, IL-8, and TNF-α^25–27^. Indeed, in our *in vitro* culture of human myometrial cells, we observed that exposure to MRS broth alone potentiated the secretion of IL-6, a hallmark pro-inflammatory cytokine. Moreover, exposure of cells to low concentrations of MRS resulted on significantly reduced IL-6 secretion in response to LPS, and exposure to high concentrations of MRS suppressed LPS-induced secretion of IL-8 and MCP-1.

The impact of MRS broth on cytokine secretion in myometrial cells represents a confounding factor in defining the effects of probiotic secretions. Thus, it was necessary to delineate the independent contribution of compounds secreted by GR-1 from those in the broth. Using PBS-based GR1SN, we found that prior exposure of hTERT-HM cells with GR1SN was required for suppression of LPS-induced cytokine secretion. As found in previous *in vivo* studies^9^ in which concurrent exposure to GR1SN and LPS failed to prevent LPS-induced PTB^9^, concurrent treatment with GR1SN-PBS did not impact LPS induction of cytokine expression. Together, these data suggest a novel hypothesis that the active components of GR1SN do not function by direct inhibition of LPS, such as effects seen with exposure to a functional inhibitor of LPS (e.g., medroxyprogesterone acetate (MPA)^18^), but rather offer immune protection to target tissues by altering intracellular pathways responsible for immune regulation.

We decided to probe two potential mechanisms that might underlie the protective action of GR1SN. First, we speculated that GR1SN preferentially activates anti-inflammatory pathways to increase secretions of anti-inflammatory cytokines, such that pro-inflammatory signals induced by subsequent LPS stimulus would be neutralized. Indeed, exposure of human placental trophoblast, mouse decidua, myometrium, placenta, and human peripheral leukocytes to GR1SN have resulted in increased secretion of anti-inflammatory cytokines such as IL-4, IL-10^8,9^ or growth factor G-CSF^12,13,28^. However, we did not observe any increase in anti-inflammatory secretion by human myometrial cells exposed to GR1SN (data not shown). Thus, we explored a second potential mechanism, namely, that GR1SN might desensitize cellular receptors to a subsequent LPS exposure.

We showed that pre-treatment of hTERT-HM cells with low-dose Pam3CSK4, a synthetic agonist of the TLR1/2 dimer complex, suppressed the induction of pro-inflammatory cytokines in response to LPS-stimulation, in a manner similar to that of GR1SN (Figs 5 and 6). This suggests that endotoxin tolerance may explain the lack of effect of LPS following GR1SN exposure. Thus, prior stimulation of TLR2 and TLR4 receptors with low dose agonists leads to their desensitization to subsequent stimulation.

TLRs play a key role in the initiation and regulation of inflammation and parturition. Thus, not only do TLR4 and TLR2 mRNA and protein increase in the myometrium at term, but TLR4 null mutant mice are resistant to infection-induced PTB^18,19^. Tolerance of TLR2^14^, TLR4^29^ and TLR9^30,31^ in human and murine immune cells reduces the expression of pro-inflammatory cytokines such as TNF-α, IL-1β, and IL-6, without increasing expression of anti-inflammatory cytokines like IL-10^14^.

Depending on the specific “TLR-agonist pair”, endotoxin tolerance operates through differential mechanisms^28,32^. Homo-desensitization, which refers to tolerance following repeated stimulus of TLR4, results in decreased surface expression of the TLR4/MD2 complex^14,33^, as well as reduced expression of adaptor proteins MHP-II and CD86^34^ at the cell membrane, without inducing IL-10 secretion in murine macrophages. Human monocyte-like THP-1 cells desensitized by LPS show alterations in the NF-kB pathway at downstream targets, reducing activation of ERK1/2 and p38^35^. However, TLR2-mediated TLR4 desensitization (hetero-desensitization) does not reduce TLR4/MD2 expression at the cell surface of murine macrophages, but can impair NF-kB dimer formations and JNK activation, leading to reduced pro-inflammatory cytokine secretion^14^. Furthermore, increased expression of regulatory molecules involved in immune activation such as TOLLIP, SOCS1, SOCS3, as well as associated suppression of pro-inflammatory pathways^36^, has also been reported as a potential mechanism of endotoxin tolerance in human intestinal cells (Caco-2) following *Lactobacillus plantarum* treatment.

Based on our findings using a low-dose Pam3CSK4 pre-treatment, it appears that the functional component of GR1SN may bear structural similarities to agonists of TLR1/2. Pam3CSK4 bears structural characteristics of bacterial lipoproteins and presents an attractive target for future research. Generally produced as pre-pro-lipoproteins, lipoproteins mature through lipidation to their functional form which can be recognized by host TLR2 receptors^37^. In gram-positive species, they are secreted in extracellular vesicles which then bind to host receptors, or are found anchored on lactobacilli cell wall^37^. Their structure, size, and function varies dependent on the bacterial species from which they are produced, which might explain the differential effects of the SNs produced by five species of *Lactobacilli*. For example, the genome of *Lactobacillus johnsonii* contains sequences for two lipoproteins, one of which may activate CD4+ T-cells, and another which has a similar composition to saliva-binding proteins found in *S*. *sanguis*^38^.

Interestingly, pre-treatment with FSL-1, a synthetic agonist for TLR2/6, did not result in suppression of LPS-induced cytokine secretion (data not shown), leading us to speculate that GR1SN pre-treatment induces endotoxin tolerance through TLR1/2 signalling in human myometrial cells by unknown lipoproteins expressed exclusively by L. *rhamnosus GR-1*.

In this study, we have successfully identified the contribution of active components secreted by probiotic *GR-1* bacteria. Our analysis of five *Lactobacilli* bacterial species highlights the importance of bacteria-tissue specificity and suggests that secretion of a TLR ligand, unique to *GR-1* bacteria, influences myometrial immune response. We have begun to strategically delineate the immune pathways that are impacted by *GR-1* bacteria products. Future research is required to define structural and functional differences between ligands expressed by *GR-1* and those produced by other bacterial species. It will also be imperative to understand the impact of endotoxin tolerance and TLR desensitization using *in vivo* and *in vitro* models. Overall, our study has revealed complexity of the myometrial response to infections. At least for *L. rhamnosus*, its probiotic effects may be more linked to desensitization at the TLR receptor than direct antagonism of infectious pathways leading to PTB.

In conclusion, we detected unique protein-like factors secreted by *L*. *rhamnosus GR-1* that suppress infection-induced myometrial cell responses. We suggest that GR1SN-mediated endotoxin tolerance dependent on TLR1/2 as a potential mechanisms to explain this suppression. We emphasize the specificity of host-microbiome interactions by demonstrating probiotic *GR-1* as a unique prophylactic agent capable of suppressing LPS-induced cytokine secretions. Future *in vitro* studies are required to isolate and purify the active components of GR1SN which may then be used *in vivo* to prevent LPS-induced PTL in animal models of infectious PTB.

## Materials and Methods

### Bacterial supernatant collection

Five Lactobacilli bacterial species, *L*. *rhamnosus GR-1*, *L*. *rhamnosus GG*, *L*. *lactis*, *L*. *casei*, *and L*. *reuteri RC-14* were independently cultured in anaerobic settings at 37 °C for 48 h on MRS agar plates and streaked for purity. Single colonies were transferred to MRS broth (55 g powder/1 L sterile H_2_O) and incubated at 37 °C for 12.5 h to mid-exponential phase (*GR-1*, OD_620 nm_ ~0.9 representing ~10^8^–10^9^ cfu/mL of bacteria). Cultures were centrifuged at 4000 g for 10 min at 4 °C to separate bacterial pellet and MRS-based supernatant (MRS-GR1SN). Bacterial pellet was washed to remove MRS broth components and transferred to PBS for a subsequent 2 hours for the collection of PBS-based GR1SN. Both MRS- and PBS-based GR1SN was centrifuged at 4000 g for 10 min at 4 °C and twice passed through 0.22μm pore filters for removal of residual bacteria.

### Primary human cells

Five primary myometrial cell lines were isolated from biopsies collected from term not-in-labour women as described earlier^39^.

### hTERT-HM cell culture

Primary human myometrial cells or myometrial smooth muscle cell line immortalized with human telomerase reverse transcriptase (hTERT-HM) were seeded into 6-well plates (100,000 cells/well) in phenol red-free DMEM/F-12 media (Gibco, ThermoFisher Scientific, MA, USA) supplemented with 10% FBS (Wisent, Bio Products, QC, Canada) and 1% Penicillin/Streptomycin/Amphoterin B (Gibco) until 60% confluent, then serum-starved in serum-free media (SFM: DMEM/F-12 supplemented with 1% ITS-A (Gibco), and 1% Pen/Strep with Amphoterin B) to synchronize cell cycles. After 8 hours in SFM, cells were pre-treated with a vehicle (MRS broth or PBS) or GR1SN for 16 hours, followed by 8 hours treatment of vehicle (PBS) or LPS (Sigma-Aldrich, Thermo-Fisher, Israel) to induce inflammation (Supplemental Fig. 2). Conditioned media (CM) were collected and protein concentration of multiple secreted cytokines was analyzed by Luminex assay or ELISA.

### Enzyme Linked Immunosorbent Assay

Sandwich enzyme-linked immunosorbent assays (ELISA) were used to determine specific cytokine concentrations in CM following various treatments. Ready-SET-Go! ELISA kits for human IL-6 and IL-8 were purchased from eBioscience (California, USA). DuoSET® ELISA kits and auxiliary kits for human MCP-1, and G-CSF were purchased from R&D Systems Inc. (MN, USA). Conditioned media were diluted 1:10 for IL-6 and IL-8, and 1:5 for MCP-1 assays using diluent solutions supplied by the manufacturer to ensure the absorbance reading would remain in range of the standard curve. Absorbance readings were conducted using μQuant^TM^ software (BioTek® Instruments, VT, USA) according to the manufacturer’s instructions.

### Luminex protein assay

Secreted cytokine, chemokine, and growth factor protein concentrations in CM were determined using a human 10-plex Bio-Plex Multiplex Cytokine Immunoassay (Bio-Rad Laboratories, Ontario, Canada). The assay measured concentrations of IL -6, IL-8, MCP-1, TNF-α, IFN-γ, IL-2, IL-4, IL-10, G-CSF, and GM-CSF. Data are analyzed using Bio-Plex Manager (version 5.0, Bio-Rad) and results are presented as concentrations in pg/mL.

### TLR-agonist kit

A human TLR-agonist kit (InVivogen, CA, USA) was used to individually stimulate TLR1-TLR10. Dose-response experiments were conducted by exposing hTERT-HM cells to a range of concentrations of individual agonists for 8 hours following manufacturer’s recommendations. Pam3CSK (TLR1/2 agonist) was tested at 0.01, 0.1, 0.5, and 1.0 µg/mL; LPS-EK (TLR4 agonist) was tested at 1, 10, 100, 1000 ng/mL; FSL-1 (TLR2/6 agonist) was tested at 1, 10, 100, 1000 µg/mL.

### Statistical analysis

All statistical analyses were carried out using PRISM software (version 5, GraphPad, CA, USA). Cytokine concentrations were compared between treatment groups and negative or positive controls using One-way ANOVA followed by Dunnett post-test. Data are expressed as mean values ± SEM. P < 0.05 was considered statistically significant. (*p < 0.05; **p < 0.01).

## Supplementary information

Dataset 1
