# Supplementary material for: Probiotic Lactobacillus rhamnosus GR-1 is a unique prophylactic agent that suppresses infection-induced myometrial cell responses
Source: Sci Rep. 2019 Mar 18;9:4698. doi: 10.1038/s41598-019-41133-0 (PMC6423128; doi:10.1038/s41598-019-41133-0)
Supplement: Supplementary file 1 — Dataset 1 [file 41598_2019_41133_MOESM1_ESM.docx]

**Manuscript** **Title**: **Probiotic *Lactobacillus rhamnosus GR-1* is a unique prophylactic agent that suppress infection-induced myometrial cell responses**

**Authors**: Bona Kim, Oksana Shynlova, Stephen Lye


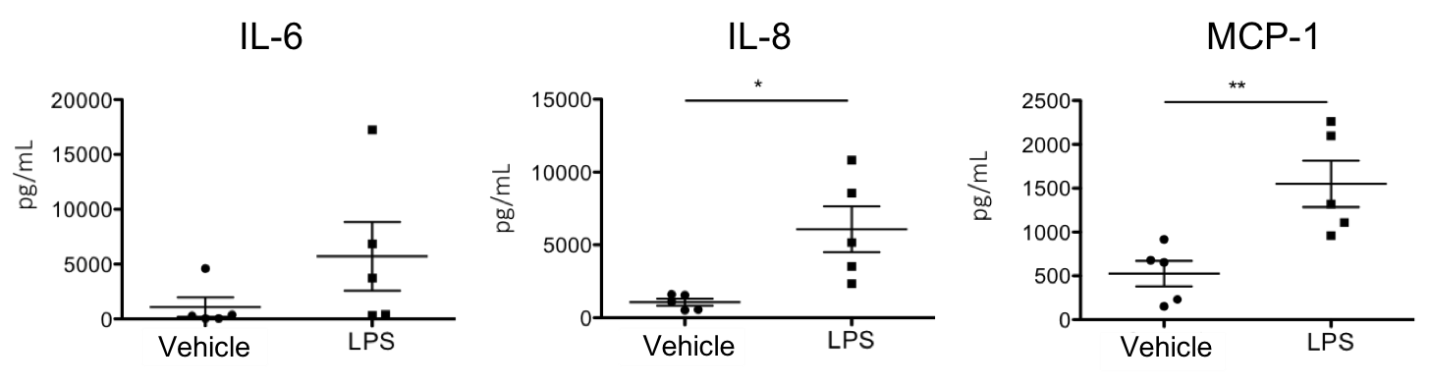


**Supplementary Figure 1.** Conditioned media from vehicle-treated (HBSS) cells and cells treated with LPS (100ng/mL) for 24 hours were analyzed for cytokine concentrations using a Luminex human 9-plex cytokine assay. Secreted cytokine concentrations are shown as absolute concentrations in pg/ml. Individual dots represent different patients (n=5). Statistical significance was determined by individual t-tests (*P<0.05; **P<0.01).

**
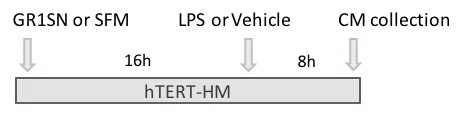
**

**Supplementary Figure 2.** Schematic of hTERT-HM treatments. GR1SN (*L. rhamnosus GR-1* supernatant) or SFM (serum-free media, negative control) were added to cell culture for 16 hours prior to LPS (lipopolysaccharide) or vehicle (PBS, phosphate-buffered saline). Cells were left to incubate for another 8 hours after which CM (conditioned media) were collected for future analysis.
